# Analyzing Functional Pathways and constructing gene-gene network for Narcolepsy based on candidate genes

**DOI:** 10.7150/ijms.41812

**Published:** 2020-06-15

**Authors:** Hui Ouyang, Zechen Zhou, Qiwen Zheng, Jun Zhang

**Affiliations:** 1 Department of Neuromedicine, Peking University People's Hospital, Beijing, China.; 2 Department of Epidemiology and Biostatistics, School of Public Health, Peking University Health Science Center, Beijing, China.

**Keywords:** single-nucleotide polymorphisms, candidate gene, hypothalamic neurons, neurodegeneration, neurology

## Abstract

**Aims:** To investigate the interactions among narcolepsy-associated genes and reveal the pathways these genes involved through bioinformatics analyses.

**Methods:** The study was performed with the following steps: 1) Selected the previously discovered narcolepsy risk genes through literature review, 2) pathway enrichment analysis, and construction of gene-gene and protein-protein interaction (PPI) networks for narcolepsy.

**Results:** 1) GO analysis revealed the positive regulation of interferon-gamma production as the most enriched terms in biological process, and C-C chemokine receptor activity as the most enriched term in molecular function, 2) KEGG pathway enrichment analysis revealed selective enrichment of genes in cytokine-cytokine receptor interaction signaling pathways, and 3) five hub genes were identified (*IFNAR1, IL10RB, DNMT1, TNFSF4* and *NFATC2*).

**Conclusion:** The bioinformatics results provide new insights into the molecular pathogenesis of narcolepsy and the identification of potential therapeutic targets for narcolepsy treatment.

## Introduction

There are two challenges in the management of narcolepsy: 1) the mechanisms of the onset and development of narcolepsy are unclear and 2) the current therapies for narcolepsy are limited, being mostly symptom-based.

Recently, the genetic risk of narcolepsy was evaluated based on the presence of HLA-DQB1*06:02, which represents an important but imperfect predictor of narcolepsy 1, 2, In addition to HLA-DQB1*06:02, many other genes have been identified as narcolepsy risk genes in international SNP-based GWASs. However, the effects of single SNPs identified by GWAS are usually small and of limited clinical significance, for both evaluating the narcolepsy risk and revealing the relevant mechanisms. The interactions among genes have not been investigated yet. Several studies investigated the benefits of conflating genomic risk estimates obtained from SNP genotyping into a genetic risk score (GRS) to predict the risk of diseases 3-7. In addition, gene networks have been studied widely for illustrating the interactions among genes 8 and the inference of biological mechanisms 9.

The pathogenesis of narcolepsy is an urgent and needs to be revealed. However, since narcolepsy is a rare disease, related GWAS research is limited, and other data are even more scarce and difficult to obtain. Because of the incomplete data, we are not able to do deep analysis of data as that in other common diseases such as stroke, diabetes and so on. However, we still need to build prediction tools and explore the pathophysiological mechanisms of narcolepsy. In order to complement the GWASs and uncover the “missing heritability”, we selected the SNPs in recently reported narcolepsy susceptibility loci (including previously reported variants in European and Asian ancestry in case-control studies), and investigated the interactions among the candidate genes, followed by pathway enrichment analysis and construction of gene networks for narcolepsy, to reveal the potential pathways and mechanisms associated with narcolepsy.

## Methods

### Genetic loci selection

We selected previously discovered narcolepsy risk loci from GWAS studies published before September, 2019 11-20. Both the narcolepsy susceptibility loci identified in Chinese population and European population were selected. The final list of candidate genes is shown in Table 1.

### Statistical Analyses

#### Go term and KEGG pathway analysis

The online software, Database for Annotation, Visualization and Integrated Discovery (DAVID, https://david.ncifcrf.gov/) 6.8 knowledgebase 22 and Retrieval Interacting genes (STRING) 10.0 platform (http://string-db.org/) 23, were used to perform Gene Ontology (GO) analysis and Kyoto Encyclopedia of Genes and Genomes (KEGG) pathway enrichment analysis, with a threshold of *p* value less than 0.05. The top 10 enrichment GO terms and KEGG pathway annotations in our study were listed. The histograms were plotted using MS Excel 2007.

#### Construction of gene and PPI networks, and hub gene analyses

The Search Tool for the Retrieval of Interacting genes (STRING) 10.0 platform (http://string-db.org/), an online tool for the structural and functional analysis of protein interactions 23, was used to obtain the interactive relationships among the candidate genes, which were further constructed using Cytoscape software 3.7.1 (http://www.cytoscape.org/) 24. The plugin cytoHubba 25 was used to select the hub genes from the PPI network. As the number of the candidate genes was limited, the cut-off criteria included a combined score < 0.2 and a node degree of > 7 for the screening of hub genes from the candidate genes. The Molecular Complex Detection (MCODE; version 1.31) app in Cytoscape was used to analyze and identify PPI network modules. Among the selected genes, the gene-gene interaction network comprising the narcolepsy-genes as nodes was constructed.

### Data availability

All experimental data within the article and its supplementary information are available from the corresponding author upon reasonable request.

## Results

### The narcolepsy-associated genes reported in previous researches

Eighteen previously reported narcolepsy-associated genes were selected, both the narcolepsy susceptibility loci identified in Chinese population and European population were selected. The candidate genes were shown in Table 1.

### GO biological process analysis and KEGG pathway enrichment of the candidate genes

**Figure 1:**
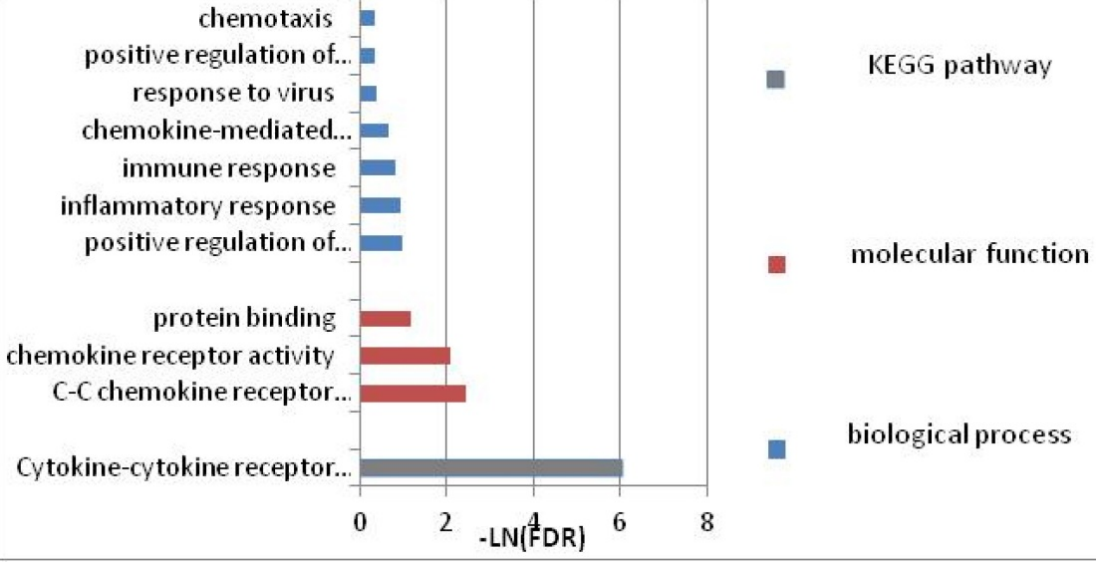
GO terms and pathways the narcolepsy-associated genes involved.

**Figure 2:**
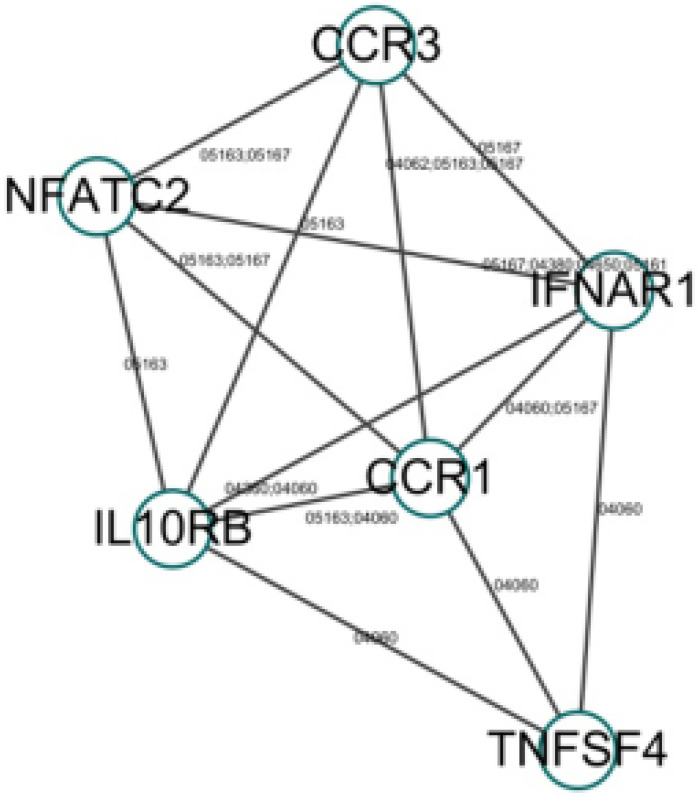
Gene-gene interaction network (the number next to the edge is the path number in the KEGG database).

To further investigate the function of these candidate genes, we uploaded these 16 genes into DAVID and STRING for GO and KEGG pathway analyses. The results indicated that the genes were mainly enriched in the pathways associated with immune response. GO biological process analysis found that in terms of molecular function (MF), the genes were associated mainly with C-C chemokine receptor activity, C-C chemokine receptor activity, chemokine receptor activity, and protein binding. In terms of biological process (BP), the genes were associated mainly with positive regulation of interferon-gamma production, inflammatory response, and immune response. KEGG pathway analysis indicated that the relevant genes were enriched in Cytokine-cytokine receptor interaction signaling pathways. Within each of the functional groups, the enrichment terms for the candidate genes with *p*-value < 0.05 are listed in Table 2. The KEGG pathways and gene-gene interaction network are depicted in Figure 1 and Figure 2.

### PPI network integration and selection of hub genes

**Figure 3:**
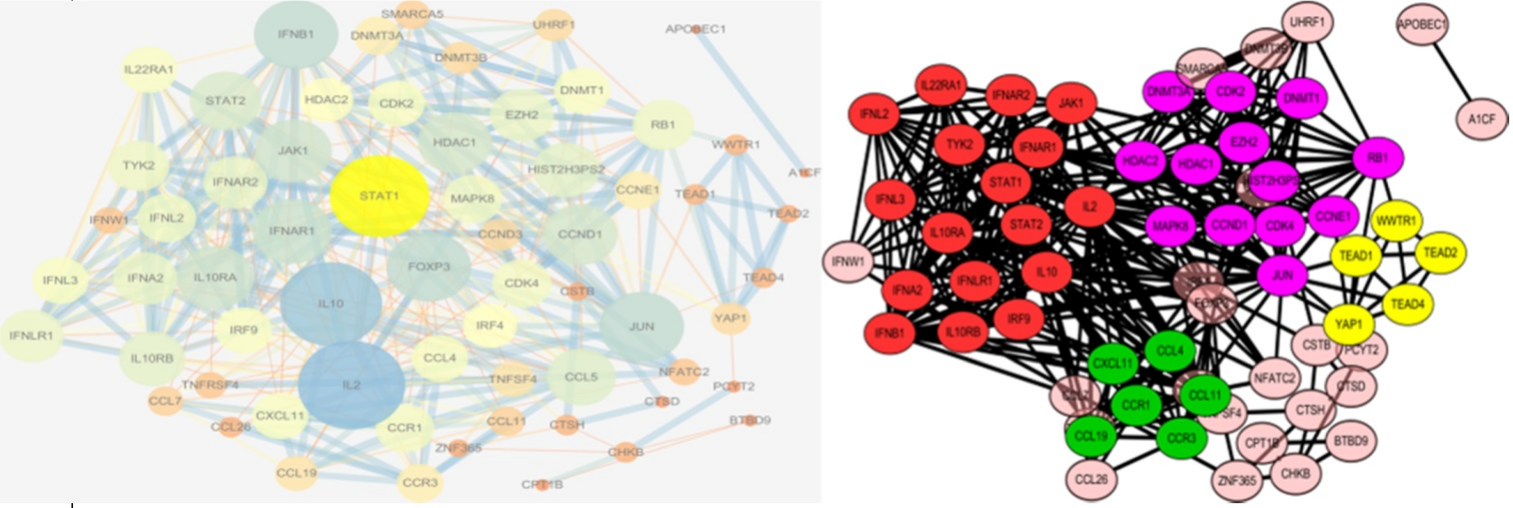
The PPI network with the degree of genes is present as the size of circles, and the combined scores between proteins are presented as the size of the lines (left). The PPI network was constructed and formatted with genes separated into four notable functional modules, in different clusters with different colors(right). PPI, protein-protein interaction.

We used the STRING database and Cytoscape to investigate PPI networks. The top five hub genes were identified among the candidate genes, as well as among all the genes in the PPI network, using cytoHubba, and the genes with the highest degrees were considered as hub genes. The key genes in the PPI network among the candidate genes are shown in Table 3. These genes may play vital roles in the onset and development of narcolepsy. The PPI networks were further analyzed using the plug-in MCODE to detect potential modules, where four notable functional modules were detected using Cytoscape software (Figure 3). The top three modules were enriched mainly in cytokine-cytokine receptor interaction, protein binding, and chemokine-mediated signaling pathways, all of which were associated with immune response.

## Discussion

Presently, the practices used for prevention, diagnosis, and treatment of narcolepsy are far from satisfactory. Exploring the genetic and molecular level dysfunction in narcolepsy patients can help develop effective treatment strategies and provide novel predictive and diagnostic clues for narcolepsy.

The results of pathway enrichment, gene-gene network, and the PPI network analyses indicated that immune responses and inflammatory responses are the main components of narcolepsy pathogenesis. These finding highlight the feasibility of developing polygenetic methods for drug development, pathogenesis exploration, and prognosis.

In our study, the GO term and KEGG pathway analyses indicated that the narcolepsy-associated genes were mainly enriched in pathways associated with immune response. GO biological process analysis found that in terms of molecular function (MF), the genes were associated mainly with C-C chemokine receptor activity, chemokine receptor activity, and protein binding. CCR (Chemokine receptors) are cytokine receptors found on the surface of certain cells that interact with a type of cytokine known as chemokine. Nineteen distinct chemokine receptors have been described in mammals, each of which has a 7-transmembrane (7TM) structure and couples to a G-protein for signal transduction within a cell, making them members of a large protein family of G protein-coupled receptors. Interaction with their specific chemokine ligands, chemokine receptors, triggers cell responses, including the onset of chemotaxis, which traffics the cell to a desired location within the organism. Some specific chemokine receptors are associated with viral affection or contribute to inflammatory diseases.

In terms of BP, the genes were mainly associated with positive regulation of interferon-gamma production, inflammatory response, and immune response, congruent to the pathways identified in the molecular function term. Interferon-gamma is a dimerized soluble cytokine that is the only member of the type II class of interferon, also known as macrophage-activating factor. It is a central regulator of the immune response and signals via the Janus Activated Kinase (JAK)-Signal Transducer and Activator of Transcription (STAT) pathway, and has broader roles in the activation of innate and adaptive immune responses to viruses and tumors. In addition, it is associated with interferon-gamma-mediated expression activation of major histocompatibility complex (MHC) class II transplantation antigen. Our KEGG pathway analysis revealed that a high level of enrichment in the cytokine-cytokine receptor interaction signaling pathways. Cytokines are soluble extracellular proteins or glycoproteins that are crucial intercellular regulators and mobilizers of cells engaged in innate and adaptive inflammatory host defenses, cell growth, differentiation, cell death, angiogenesis, and development and repair processes aimed at the restoration of homeostasis. Cytokines are released by various cells in the body, usually in response to an activating stimulus, and they induce responses through binding to specific receptors on the cell surface of target cells.

The results of the functional enrichment analyses demonstrated that the narcolepsy-associated genes were enriched in immune-related pathways. This is consistent with previously proposed hypothesis that the immune system plays a key role in narcolepsy 36, 37, based mainly on the indirect evidence of the close association between narcolepsy risk with H1N1 and HLA gene. However, the exact underlying mechanisms of this association remain unknown 37, 38. The enrichment of narcolepsy-associated genes in C-C chemokine receptor activity, chemokine receptor activity, interferon-gamma production, and cytokine-cytokine receptor interaction pathways indicated that the onset of narcolepsy is associated not only with the cross-reaction autoimmune processes, but also with the dysfunction of immune-regulation, especially the dysfunction of cellular immunity. These results can help provide clues to reveal the exact mechanism of immune system dysfunction in narcolepsy patients, and possibly help identify new targets for immune therapy of narcolepsy, such as immune-modulation to prevent or delay the progression of narcolepsy.

We also constructed a PPI network and identified the top five hub genes, i.e., IFNAR1, IL10RB, DNMT1, TNFSF4, and NFATC2. Our results indicated that IFNAR1 was at the core of the PPI network. It belongs to the type II cytokine receptor family and encodes a type I membrane protein that forms one of the two chains of the type I interferon receptor. Binding and activation of the receptor leads to the phosphorylation of several proteins, including STAT1 and STAT2. The encoded protein also functions as an antiviral factor; it transforms the cell to an antiviral state in coordination with other cytokines 39, 40. Various studies have reported that the pathways associated with IFNAR1 are associated with several diseases caused by viral affection 41-44. It has been reported that the onset of narcolepsy is associated with H1N1 infection 45, 46. It provides indirect evidence that the onset and progression of narcolepsy may be associated with abnormal immune response following a virus infection, in which the IFNAR1 dysfunction likely plays an important role, finally leading to the destruction of hypocretin neurons due to immune attack. The results indicated that the immune dysfunction of narcolepsy is associated not only with abnormal antigen recognition and cross-reaction, as indicated by the association of the HLA-DQB1*06:02 and TRA with narcolepsy risk 13, 20, 47, but also with the dysfunction of IFNAR1 immune-modulation and anti-viral processes. Thus, IFNAR1 may serve as a novel target for immune therapy of narcolepsy in the future.

Based on this analysis, the PPI network was identified and divided into four groups according to the protein-protein interactions. The top module included genes such as IFNAR1, IL2, and IL10RB, which are enriched mainly in the cytokine-cytokine receptor pathway. This result is in accordance with the results of the pathway enrichment analysis, and supported the role of IFNAR1 as the hub gene in the PPI network.

Nevertheless, the present study has some limitations. First, non-genetic factors may contribute to the onset of narcolepsy, and the effect of gene-environment interactions on narcolepsy incidence should also be considered. Second, the biological verification was not involved in the study. So further experimental studies are needed to confirm the identified hub genes and pathways, including RT-qPCR validation of these hub genes in clinical samples.

## Conclusion

Through a comprehensive bioinformatic and heredity statistics re-analysis of GWAS data, we identified some crucial genes and pathways that were closely correlated with narcolepsy. Therefore, an overview regarding the molecular pathogenesis of narcolepsy and the potential for identification new drug targets for narcolepsy was provided. The results reported in the present study can eventually offer novel clues for screening and clinical diagnosis of narcolepsy, as well as understanding the molecular mechanisms underlying the pathogenesis of narcolepsy. However, further molecular biological experiments are required to verify these findings.

## Figures and Tables

**Table 1:** The selection of SNPs

Chr	Gene	SNP	Tag SNP	Allele	*Risk Allele*
1	TNFSF4	rs7553711	rs4090391	C/T	T
1	MIR-552	rs10915020	rs12026171	A/G	G
3	CCR1	rs3181077	-	C/T	C
3	CCR3	rs3181077	-	C/T	C
4	BTBD9	rs3923809	-	A/G	G
7	TRB	rs2854536	-	T/C	T
8	UBXN2B	rs2859998	rs6993992	T/C	T
10	ZNF365	rs10995245	-	G/A	A
10	A1CF	rs4290173	rs16911668	A/G	A
12	TEAD4	rs12425451	rs12322530	G/A	G
14	TRA	rs1154155	-	T/G	T
14		rs12587781	rs1154153	T/C	T
14		rs1263646	rs1263647	A/G	A
15	CTSH	rs12148472	-	T/C	C
15		rs3825932	-	T/C	T
19	DNMT1	rs2290684	rs2114724	C/T	T
19		rs6511570	rs7253062	G/A	G
20	NFATC2	rs8119787	-	A/G	G
21	IL10RB	rs2834118	rs2834113	T/G	G
21	IFNAR1	rs2252931	rs2834188	A/G	A
22	CPT1B	rs5770917	rs5770911	C/T	C
22	CHKB	rs5770917	rs5770911	C/T	C

**Table 2:** Signaling pathways enriched by the candidate genes

Category	Term	Gene count	Background number	p-value	FDR
*KEGG pathway*					
hsa04060	Cytokine-cytokine receptor interaction	5	243	2.70E-04	0.002418919
*GO:molecular function*					
GO:0016493	C-C chemokine receptor activity	2	12	9.91E-03	0.091466094
GO:0004950	chemokine receptor activity	2	17	0.014012135	0.127079826
GO:0005515	protein binding	12	8785	0.040126286	0.325943103
*GO:biological process*					
GO:0032729	positive regulation of interferon-gamma production	2	46	0.037690464	0.375903573
GO:0006954	inflammatory response	3	379	0.038655183	0.383537965
GO:0006955	immune response	3	421	0.046770989	0.444448439

**Table 3:** The key genes in the PPI network, among the candidate genes

Gene	Description	Function	Degree (score)
IFNAR1	interferon alpha and beta receptor subunit 1	Encodes a type I membrane protein that forms one of the two chains of a receptor for interferons alpha and beta.	23
IL10RB	interleukin 10 receptor subunit beta	Encodes an accessory chain essential for the active interleukin 10 receptor complex.	18
DNMT1	DNA methyltransferase 1	Encodes an enzyme that transfers methyl groups to cytosine nucleotides of genomic DNA.	14
TNFSF4	TNF superfamily member 4	Encodes a cytokine of the tumor necrosis factor (TNF) ligand family.	10
NFATC2	nuclear factor of activated T cell 2	A DNA-binding protein with a REL-homology region (RHR) and an NFAT-homology region (NHR).	7
